# A Critical Review of the Support for Variability as an Operant Dimension

**DOI:** 10.1007/s40614-020-00262-y

**Published:** 2020-07-27

**Authors:** Siv Kristin Nergaard, Per Holth

**Affiliations:** Faculty of Health, Institute for Behavioral Science, OsloMet – Oslo Metropolitan University, St. Olavs Plass, PO Box 4, 0130 Oslo, Norway

**Keywords:** Direct reinforcement of variability, Molar, Molecular, Reinforcement, Extinction, Descriptive and functional classes

## Abstract

There is abundant evidence that behavioral variability is more predominant when reinforcement is contingent on it than when it is not, and the interpretation of direct reinforcement of variability suggested by Page and Neuringer, Journal of Experimental Psychology: Animal Behavior Processes, 11(3), 429–452 (1985) has been widely accepted. Even so, trying to identify the underlying mechanisms in the emergence of stochastic-like variability in a variability contingency is intricate. There are several challenges to characterizing variability as directly reinforced, most notably because reinforcement traditionally has been found to produce repetitive responding, but also because directly reinforced variability does not always relate to independent variables the same way as more commonly studied repetitive responding does. The challenging findings in variability experiments are discussed, along with alternative hypotheses on how variability contingencies may engender the high variability that they undeniably do. We suggest that the typical increase in behavioral variability that is often demonstrated when reinforcement is contingent on it may be better explained in terms of a dynamic interaction of reinforcement and extinction working on several specific responses rather than as directly reinforced.

In ontogenetic selection, as well as in phylogenetic selection of behavior, the adaption to changing contingencies is explained parsimoniously by the principles of variation and selection (e.g., Donahoe & Palmer, 2004). Sources of variation are important both for theoretical and for practical reasons. Without continued sources of variation, selection alone could only serve to further constrict variation within preexisting boundaries. Thus, any evolution of species or of behavioral repertoires beyond preexisting characteristics depends on continuous sources of variation. For practical reasons, sources of behavioral variation are important because they can help us increase or decrease variability when such changes are desirable or essential (Neuringer, 2002).

In an extensive article, Page and Neuringer (1985) suggested that variability may be an operant dimension of behavior, in the same way as, for example, response rate, force, duration, and location. Several later experiments have shown that variability, normally measured as the dispersion of different sequences, are higher when a variability contingency is in effect, than when it is not (e.g., Denney & Neuringer, 1998; Kong, McEwan, Bizo, & Foster, 2019; Miller & Neuringer, 2000). This finding is highly reliable, and the interpretation of “direct reinforcement of variability” and “variability as an operant dimension” has been widely accepted (e.g., Catania, 2013; Doughty & Galizio, 2015; Dracobly, Dozier, Briggs, & Juanico, 2017; Lee, Sturmey, & Fields, 2007; Locey & Rachlin, 2013; Odum, Ward, Barnes, & Burke, 2006; Rodriguez & Thompson, 2015; Stahlman & Blaisdell, 2011; Stokes, 1995; Stokes, Mechner & Balsam, 1999; Ward, Kynaston, Bailey, & Odum, 2008). Nevertheless, a few researchers (Barba, 2015; Epstein, 2014; Holth, 2012; Machado, 1989, 1992, 1993, 1997; Machado & Tonneau, 2012; Marr, 2012) have raised the question of whether direct reinforcement of variability is the most satisfactory view or whether the variability in these experiments is more effectively or pragmatically considered as a derivative of other procedural characteristics.

The aim of the present article is to discuss whether directly reinforced operant variability is a satisfactory description of the behavioral variability that results from a variability contingency. In addition, we question the common notion of uncertainty and unpredictability (Jensen, Miller, & Neuringer, 2006; Neuringer & Jensen, 2013; Page & Neuringer, 1985) of the resulting variable responding. First, we summarize the basis provided by Neuringer and colleagues for the commonly accepted interpretation of variability as directly reinforced, as well as alternative interpretations, most notably by Machado and by Barba. Second, we will present results of previous studies where variability considered as an operant dimension has been found to differ from other, typical operant dimensions. Third, we describe some potentially important procedural details. Fourth, based on Catania’s (1973, 2013) analysis of the concept of the operant in terms of descriptive and functional classes we will discuss whether the descriptive unit commonly used in variability experiments can be assumed to turn into a valid functional unit. Fifth, we contrast a molar and a molecular interpretation of variability. Finally, an alternative interpretation where variability is interpreted as a derivative phenomenon instead of a selected dimension of directly reinforced operant behavior is suggested as a more parsimonious explanation of the results that have been consistently replicated in experiments using variability contingencies. In this regard we have two main concerns. The first is with specific details of the mainstream way of conducting variability experiments. Our second concern is the extensive use of aggregated statistics when interpreting results. Aggregated statistics are calculated from steady-state behavior, which does not necessarily include data relevant to the identification of functional relations in the development of responding (Reid, Chadwick, Dunham, & Miller, 2001; Shimp, 2013; Skinner, 1976). We argue that in order to arrive upon a solid interpretation of the behavioral variability that results when reinforcement is contingent upon it, experimental procedures that allow us to keep track of functional units are needed.

## The Support for Behavioral Variability as an Operant Dimension

Page and Neuringer (1985) demonstrated that a variability contingency could result in varied sequences of pecks in pigeons, and Neuringer (1986) showed that human participants could learn to distribute two different responses randomly—according to an extensive set of statistical criteria. Neuringer and Jensen (2012) listed three traditionally mentioned causes of behavioral variability and added a fourth: (1) behavior caused by unrelated events (in the environment or within the organism); (2) simply unexplained behavior; (3) behavior induced by extinction or aversive events; and (4) directly reinforced variability. Neuringer’s hypothesis focuses on the fourth source: directly reinforced variability. According to Neuringer and Jensen (2012) variability is selected when reinforcement is contingent on it. Hence, varying between sequences is considered as being directly reinforced and functioning as an operant dimension on its own: “Stated simply, variability is a reinforceable dimension of emitted behaviors” (p. 74).

Neuringer’s hypothesis is that the underlying process of this stochastic-like behavior is an inborn variability generator (Page & Neuringer, 1985) or an endogenous stochastic process (Jensen et al., 2006; Neuringer, 1986, 2002). The theory of an endogenous stochastic process suggests that memory for prior responses is not consistent with stochastic-like performance (Neuringer, 2002), because a defining feature of random and stochastic processes is a lack of predictability. Acknowledging that this underlying endogenous stochastic process can never be proven, Neuringer (2002) listed several experimental findings that are consistent with this process. These findings are from experiments where stimulus control by prior responses are in some way interfered with (Baddeley, 1966; Machado, 1993; Manabe, Staddon, & Cleaveland, 1997; Morris, 1987; Neuringer, 1991; Page & Neuringer, 1985) or the experimental findings are consistent with response distributions from a random generator (Blough, 1966; Machado, 1989; Neuringer, 1986). Even if the theory of an endogenous stochastic process underlying the variability in these experiments is satisfactory as a descriptive summary of experimental findings, it is still unclear how the mechanisms are most usefully described, when reinforcement in some cases produces stereotypy whereas in others it produces highly variable behavior.

Doughty and Galizio (2015) suggested that variability may be a generalized higher-order response class, learned from multiple exemplar training, where the response unit is not necessarily the whole sequence, but possibly two consecutive responses. In this explanation the contingency targets within-sequence variability, as opposed to what the contingency is programmed to reinforce—between-sequence variability. The variability is suggested to be directly reinforced, because it is hypothesized that the differential reinforcement teaches the subjects that extended periods of only switching between operanda or repeating the same operandum is likely to be followed by timeout (nonreinforcement), whereas reinforcement is likely to follow a mixture of these events.

## Previous Challenges to the View of Variability as an Operant Dimension

A few authors have questioned whether the results from variability experiments so far have shown direct reinforcement of variability, even if this is the commonly accepted interpretation. Machado (1993) asked what property defined the operant variability class. He suggested that identifying the necessary and sufficient conditions to engender variable responding, and describing the mechanisms or processes underlying the behavior would be necessary to answer this question. In several articles, Machado (1989, 1992, 1993, 1994; Machado & Tonneau, 2012) has argued that the necessary, although not always sufficient, condition is a negative frequency-dependent contingency. Machado’s (1993) Frequency-Dependent Hypothesis proposes that the variability in these schedules emerges or is mediated by frequency-dependent selection rather than being directly reinforced. Machado and Tonneau (2012) explained this process as “one strengthening the weakest responses and the other weakening the strongest responses are logically sufficient to promote and maintain behavioral variability” (p. 254). Adapted from Machado’s Frequency-Dependent Hypothesis is Barba’s (2015) Balance Hypothesis. According to this interpretation, a frequent response just reinforced will lose its strength because reinforcement is less likely to follow from it again, at least for some time. The least likely responses, the responses not emitted before or not emitted for a long time, will gain in strength because they are momentarily more likely to be reinforced.

Catania (1973) distinguished between descriptive and functional response classes. A descriptive class consists of responses that satisfy the reinforcement contingency, and a functional class is the class of responses generated by the contingency. An operant is characterized by a correspondence between a descriptive and a functional class. Barba’s (2015) interpretation suggests that although the descriptive unit is sequences, because reinforcers are contingent on the variability of the emitted sequence in relation to previous sequences, the functional unit is nonetheless not the whole sequence. The hypothesis does not assume a formation of a structurally consistent functional class, reinforcement is rather hypothesized to act upon properties intrinsic to the sequence, that is, not dependent on the relation to other sequences (Barba, 2015). It is important to note that in frequency-dependent selection and Balance Hypothesis variability or unpredictability is not directly reinforced. The contingency does not engender an operant class properly characterized as responding unpredictably. It is rather a constant, dynamic organism-environment interaction that produces and maintains the variable responding, because the contingency continuously balances the strength of each sequence or pattern and no specific response or response pattern is consistently selected (Barba, 2015; Machado & Tonneau, 2012). Inseparably linked to variability contingencies is the repeated cycling between reinforcement and the differential extinction of recently emitted behavior.

The important distinction between Neuringer’s and Doughty and Galizio’s interpretation on the one hand, and Machado’s and Barba’s interpretation on the other, is whether the best explanation of the data published so far is the direct reinforcement of variability or whether it is more productive to view variability as a joint and dynamic effect of reinforcement and extinction of many different responses. From the different interpretations of variability presented above, it seems clear that there are still several unanswered questions regarding the mechanisms of variable responding.

## Does Variability Behave Consistently with Other Operant Dimensions?

The standard interpretation, explained above, that variability is a directly reinforced operant dimension, should entail that such variability behaves consistently with other operant dimensions under different circumstances. However, Wagner and Neuringer (2006) contended that although variable operant responding is controlled by reinforcement contingencies, some characteristics of operant variability may differ from more typically investigated repetitive responding. First, a general finding in research on operant behavior is that, all else equal, responses closer to the reinforcer (measured by time or number of responses) conforms to the contingency faster and more closely than responses further away from the reinforcer. Experiments have shown that the longer time from the response to the delivery of a reinforcer reduces the reinforcing effect. Consult research on how temporal distance decrease the effectiveness of reinforcement (Catania, 1971; Catania et al., 2015; Killeen, 1994), and research on delayed reinforcement (e.g., Dews, 1960; Ramey & Ourth, 1971; Richards, 1981; Skinner, 1938/1991). However, in a variability contingency this relation is reversed: behavior exposed to a variability contingency comes to correspond to the contingency faster and more closely with the inclusion of a forced interresponse time (IRT, in the literature commonly denoted IRI for interresponse interval) than without (Morris, 1987, 1989; Neuringer, 1991; Page & Neuringer, 1985). Morris (1987) conducted a variability experiment with two different conditions. The first was a free-operant procedure, where key lights were lit, and key pecks were recorded continuously. The second was a discrete-trial procedure, where the key lights and houselight turned off for a forced IRT of 2 s following each individual key peck. In both conditions, reinforcement was contingent upon sequences of four key pecks distributed on two keys that differed from the two immediately preceding sequences. The free-operant and discrete-trial conditions produced surprisingly different results. During the free-operant sessions, fewer than 32% of completed sequences met the reinforcement criterion, whereas during the discrete-trial sessions with the forced IRTs approximately 75% met the criterion.

Neuringer (1991) also investigated the effects of the forced IRT, using Long-Evans rats. His results showed that in a contingency where sequences of four lever-presses had to differ from the immediately preceding five sequences without a forced IRT, variability was relatively low. In contrast, sessions with a 1-s forced IRT generated higher variability. After manipulating the IRT from 0 to 20 s, he found that the variability across sequences increased as a function of the duration of the forced IRT, as the response repetitions within-trials decreased. The increase in variability as the IRT increased from 0 to 1 s was dramatic, whereas longer IRTs only increased the variability slightly. A forced IRT is commonly used in experiments with pigeons as a response-pacing requirement, as pigeons tend to peck rapidly on a single key (Blough, 1966; Shimp, 1973). The original pacing argument for the IRT may be less valid with respect to lever pressing in rats. Yet, the forced IRT is also typically used in variability experiments with rats and regardless of the validity of its original justification, it has an equally dramatic effect on the variability of responding as in the pigeon experiments. It is still unclear why and how the IRT has such a dramatic effect on variability (cf. Doughty & Galizio, 2015).

Another area of interest is the research on units integrating several individual responses. The general finding is that the fewer responses a unit or chain consists of, the faster it will conform to the contingency. For example, a sequence consisting of two responses will more easily conform to the contingency than sequences consisting of three or four responses (e.g., Reed, Schachtman, & Hall, 1991; Schneider & Morris, 1992). As in the previous example on IRT, this relation is also reversed in a variability contingency: behavior conforms faster and more closely to this contingency the longer the descriptive unit is. For example, Page and Neuringer’s (1985) Experiment 4 investigated the influence of an increasing number of pecks in the descriptive unit, 4, 6, and 8 pecks. They concluded that increasing the number of responses per sequence resulted in an increased percentage of reinforced trials, as responding conformed more closely to the contingency. Their analysis showed that variability within the sequences did not significantly change, but as more sequences are available for reinforcement when the sequences are longer, the overall probability of sequences leading to reinforcer delivery increases.

A third finding was reported by Odum et al. (2006). They found that repetition and variability were differentially affected by prereinforcement delays. In the repeat condition longer delays between the last response and delivery of reinforcement resulted in a decrease of the targeted sequence (mostly RRLL), variability increased, and reinforcement probability decreased. In the variability condition the variability and percentage of trials ending in reinforcement typically remained unaffected by the lengthening of the delay. This effect of delay of reinforcement on variability was also shown by Wagner and Neuringer (2006). They made reinforcement contingent on either of three different levels of variability, low, medium, and high. The sessions with prereinforcement delay showed a modest increase in variability in the low and medium group, whereas variability decreased slightly in the high variability group.

These three findings in variability experiments lead to the conclusion that, the longer time between the effective responses in the sequence, or the more responses separating the early responses in the sequence and the reinforcer, the closer the behavior conforms to the variability contingency. This conclusion challenges the otherwise established generality of the delay-gradient (e.g., Catania, 2005; Catania et al., 2015; Killeen, 1994).

A fourth example of a different effect on repetition and variability is the finding that alcohol does not affect behavior significantly when responding is already variable or random-like, whereas it has a disruptive effect on maintaining repetitive behavior (Cohen, Neuringer, & Rhodes, 1990; McElroy & Neuringer, 1990). According to Neuringer and Jensen (2012) these findings listed above are consistent with their hypothesis that memory for, or discriminative control by, earlier responses are detrimental to variability and, by default, also consistent with the stochastic generator hypothesis.

A fifth finding is that variability tends to decline when a possible reinforcer approaches (Cherot, Jones, & Neuringer, 1996; Gharib, Gade, & Roberts, 2004; McElroy & Neuringer, 1990; Wagner & Neuringer, 2006). This result is in accordance with findings on repetitive behavior (Boren & Gollub, 1972; Ferster, 1960; Nevin, Cumming, & Berryman, 1963; Stubbs, 1968). When reinforcement is contingent on repetitive behavior, the behavior will commonly be even more repetitive when a possible reinforcer is approaching, increasing accuracy according to the contingency (e.g., Nevin, 1967). In a variability contingency a higher level of repetitive responding has also been found when approaching a possible reinforcer, but in this contingency, increased repetition implies a decrease in accuracy. Hence, with standard operants the properties upon which reinforcement is contingent become more likely closer to the reinforcer, whereas in a variability contingency the property upon which reinforcement is contingent, variability, becomes less likely closer to the reinforcer. McElroy and Neuringer (1990) analyzed the internal structure of the sequences in their variability experiment. They found that the last response in the sequence, the fourth response, was more likely to be a repetition of the third response than the previous responses in the sequence were likely to repeat the previous response. Cherot et al. (1996) investigated even more thoroughly the repetition-inducing effect of reinforcement. In this experiment the descriptive unit was still four lever-presses or key pecks, but the sequences were reinforced on an FR 4 schedule, meaning that only the fourth sequence that satisfied the contingency would be reinforced. The variability was higher in the first two sequences in the FR 4 schedule, and declined in the third and fourth sequence. Doughty, Giorno, and Miller (2013) found that this effect was even more predominant with larger reinforcers.

The repetition-inducing effect when a reinforcer is typically scheduled, the nonaffect of reinforcement delays, and other disrupters like alcohol, all seem to set variability aside from standard operant dimensions. In addition, the fact that behavior conforms to the contingency faster and more closely in a variability contingency with the inclusion of a forced IRT, and the longer the descriptive unit is, suggest that it is worth considering alternatives to the idea of variability as directly reinforced.

## Potentially Important Procedural Details

The following variability contingency procedures have been used: recency, frequency-dependent, and threshold contingencies. In a contingency based on recency, commonly a lag-schedule, reinforcers are contingent on a unit of responses that are not among the most recently emitted. In Lag *n*, reinforcement is contingent on a response that differs from the immediately preceding *n* responses or sequences of responses. Page and Neuringer’s (1985) Experiment 3 manipulated the lag value (*n*) from 5 to 50 using sequences of eight key pecks. The result of this experiment was that the percentage of different sequences emitted increased from Lag 5 and up to Lag 25, and then decreased slightly from Lag 25 to Lag 50.

A frequency-dependent procedure only reinforces the least likely or the least often emitted among the available responses. Frequency-dependent procedures can be either absolute, where only the response sequence that has occurred least frequently is reinforced (Blough, 1966; Shimp, 1967), or probabilistic, with a payoff probability corresponding to how infrequently a response is emitted (Machado, 1992). Blough (1966) reinforced the least-frequent interresponse time in an experiment with pigeons. He divided the interresponse times into “IRT-bins” of different durations. Whenever a response was reinforced, the bin of interresponse times with fewest occurrences was selected for the next reinforcement. The procedure resulted in highly variable interresponse times.

A third variability procedure is a threshold procedure, where the reinforced responses are those with a frequency below or equal to a specified threshold. For example, Denney and Neuringer (1998) used a threshold contingency of .09 with sequences of four key pecks. This contingency delivers reinforcement for sequences occurring less than or equal to 9% of the total trials.

The leniency of the variability contingency will affect the behavior in predictable ways. An example of a lenient variability procedure is the frequency-dependent contingency targeting individual responses used by Machado (1992). In this experiment the probability of reinforcement was calculated from previously emitted L or R key pecks. The more predominant R key pecks the less probable was reinforcement for R key pecks and vice versa. Only when left and right key pecks were evenly emitted (indifference), the probability of reinforcement was equal for both responses. A pattern of strict alternation between responses emerged. These lenient types of variability procedures are the ones used in most applied settings (e.g., Esch, Esch, & Love, 2009; Heldt & Schlinger, 2012). When more stringent variability procedures are in effect, a very different pattern of behavior is predominant. In these procedures a higher-order stereotyped pattern that would satisfy the contingency exists, but would be exceedingly long, especially in procedures where the descriptive unit consists of response sequences. Commonly responding becomes apparently stochastic- or random-like. This may be characterized as a “memory capacity problem” or as a complexity problem, in which the discriminative control by previous responses seems to break down and stochastic-like responding emerges (e.g., Holth, 2012; Machado, 1993; Page & Neuringer, 1985). According to Jensen et al. (2006), the overall relative frequency of stochastic responding can be predicted, whereas the moment-to-moment responses are independent of prior events and cannot be predicted any more precisely than chance (random), or according to a probability statement (stochastic). These stringent procedures have mostly been performed on nonhuman animals. The focus of this article is the stochastic-like responding occurring in stringent variability procedures. These are most typically found in laboratory research with nonhuman animals, but we will also discuss some findings from applied studies with human participants.

Schwartz (1980, 1981) investigated if sequences of several responses could become integrated (i.e., functional) behavioral units. In these experiments he used a five by five light matrix where pigeon’s pecks moved a light from its start position in the upper left corner either to the right or down in the matrix, in order to move the light from start to goal position in the bottom right corner. The unit was therefore any sequences of four left and four right key pecks needed to move the light from start to goal. He reported that reinforcement commonly resulted in stereotyped behavior in pigeons, even when stereotypy was not a requirement. Later, he reported that even when variability was a requirement, reinforcement would still result in stereotypy (Schwartz, 1982). According to Schwartz’s interpretation, therefore, variability and novelty could not be a product of reinforcement.

Page and Neuringer (1985) hypothesized that the constraint in Schwartz’s experiments, of exactly four pecks on either disk, was what led to stereotyped responding. In their Experiment 1, they investigated the difference of two conditions. One was a Lag 5 contingency called variability (V), the other a Lag 1 with the constraint of only four pecks on either key, a fifth peck would lead to a timeout and start a new sequence, this condition was called variability with constraint (VC). Both conditions included a forced 0.5-s IRT. This IRT, not present in Schwartz’s experiments, functioned as a short time-out between all the individual responses in the sequence, except between the last response and accessing the reinforcer. It was signaled by the darkening of lights, and responding had no programmed consequences, except resetting the interresponse time. The result of Experiment 1 showed that in the V-sessions significantly more reinforcers were obtained than in the VC-sessions, but variability, measured by the percentage of different sequences per session, were close to identical. They concluded that the constraint was responsible for the lack of success in Schwartz’s (1982) experiment. In Page and Neuringer’s (1985) Experiment 2, a direct replication of Schwartz’s (1982) Experiment 1, without the forced IRT, they again found that fewer of the available reinforcers were obtained with the constraint than in sessions without the constraint. Variability, on the other hand, was higher in the sessions with constraint. Page and Neuringer (1985) concluded from the experiment that the constraint was responsible for the low frequency of obtained reinforcers. In addition, they concluded that variability was higher in the VC condition because the variability criterion was so low, Lag 1, arguing that the low criterion was more discriminable during the V-sessions than in the VC-sessions. The possible implications of the forced IRT were not discussed, but it was implemented in the remaining experiments, and is a common variable in variability experiments to date. As a result of Page and Neuringer’s experiments, Schwartz’s results and interpretation was deemed to be flawed (Catania, 2013), and Page and Neuringer’s (1985) conclusion regarding direct reinforcement of variability and variability as an operant dimension has been commonly accepted.

Another possible distinction in the V and VC conditions with Lag 1 was in whether more than one response sequence was necessarily reinforced. In the V condition all sequences were eight responses long, and the only way to produce a timeout was to repeat, whereas in the VC-conditions both repeating a sequence and pecking a disc a fifth time would produce a timeout. In a Lag 1, without constraint, the only incorrect sequence would be the repeating of the preceding response. Hence, a different sequence had to occur and be reinforced before the first sequence again would produce reinforcement. This is not the case in the Lag 1 with constraint, VC, where the first sequence may be reinforced again after any “error” of five pecks on the same key (Schwartz, 1982). In effect the constraint of no more than four responses on each key makes possible the reinforcement of a single sequence only, so that no alternative sequence is ever reinforced. Thus, whereas the VC condition allows for intermittent reinforcement of a single response sequence, the V condition forces the alternating reinforcement of at least two different sequences. A similar difference is also present between a variability contingency and the control condition in variability experiments commonly called “yoke.”

Procedures that have been found to induce variability in behavior include slowed responding, for example, including an IRT (Morris, 1987, 1989; Neuringer, 1991), intermittent reinforcement (e.g., Eckerman & Lanson, 1969; Ferraro & Branch, 1968), delayed reinforcement (e.g., Odum et al., 2006), as well as extinction (e.g., Antonitis, 1951; Eckerman & Lanson, 1969; Iversen, 2002; Mechner, 1958; Mechner, Hyten, Field, & Madden, 1997; Notterman, 1959; Schwartz, 1980; Stokes, 1995). Several experiments have shown more variability during intermittent than during continuous reinforcement. To rule out such induced intermittent and delayed reinforcement effects, several variability experiments have included the yoke control condition. In this condition, reinforcement of sequences is based on reinforcers delivered on a previous trial or the reinforcers earned by the same or another subject in an earlier session. In effect, the yoke schedule becomes a VR-schedule of reinforcement of sequence completion, regardless of left or right position. This condition allows variability, but it does not require it. The relatively repetitive behavior in the yoke condition, compared with the variable behavior when the variability contingency is in effect, indicates that inducing effects of intermittent and delayed reinforcement are *not* the primary sources of the variability in these contingencies.

However, the yoke procedure is sometimes purported as a control procedure for the differential extinction in these experiments (Neuringer, 2002, 2004; Page & Neuringer, 1985). Extinction is a familiar source of variability. Several studies (e.g., Antonitis, 1951; Eckerman & Lanson, 1969; Iversen, 2002; Mechner, 1958; Mechner et al., 1997; Notterman, 1959; Schwartz, 1980; Stokes, 1995) have shown that variability increases initially when the contingency changes from reinforcement to extinction. Where there is extinction there may be extinction-induced variability. As concluded above, the yoke condition is a good control condition for intermittent and delayed reinforcement, as these variables are still present in the control condition. Contrary, because reinforcement is independent of sequence variability, and therefore there is no consistent or enduring extinction of any specific response: “the pigeons could vary or not without penalty” (Jensen et al., 2006, p. 460), we argue that it is *not* a satisfactory control condition for extinction-induced variability. Whereas reinforcement according to a lag schedule, for example, always requires the occurrence of different descriptive units, at least two, the yoke condition allows for intermittent reinforcement of a single unit—even without the occurrence of others.

## The Behavioral Unit: Descriptive and Functional Classes

As previously mentioned Catania (1973) distinguished between descriptive and functional classes. Moreover, as outlined by Catania (2013), in order to determine that operant reinforcement has taken place, three criteria must be fulfilled: first, certain responses must produce consequences. Second, such responses occur more often when they produce those consequences than when they do not. Third, the increased responding must occur exactly because those responses are followed by those consequences.

As Barba (2012a) pointed out the correspondence between the descriptive and the functional class, has not typically been met in experiments on behavioral variability. Whereas the descriptive class is defined by some variability contingency, the functional class is typically defined in terms of aggregated statistics. The most typically studied functional class is variability measured as the relative frequency of emitted sequences. A statistical analysis used in most variability experiments is the U value (e.g., Abreu-Rodrigues et al., 2005; Doughty & Lattal, 2001; Kong et al., 2019; Odum et al., 2006; Page & Neuringer, 1985; Ward et al., 2008), an aggregated statistical uncertainty measure (see appendix for the most commonly used formula), derived from information theory (Miller & Frick, 1949) introduced by Page and Neuringer (1985). Despite the suggestion that the U value provides indices of level of uncertainty and stochasticity (Neuringer, 2004; Neuringer & Jensen, 2013; Page & Neuringer, 1985), it does not, by itself, measure uncertainty when calculated as a single value over entire sessions (Kong, McEwan, Bizo, & Foster, 2017; Souza, Pontes, & Abreu-Rodrigues, 2012). Rather, the U value indicates a relative frequency of the available units. A U value of 0 would indicate no dispersion, only one of the available units (whether this is individual responses or sequences of several responses) have been emitted by the subject, indicating a completely peaked frequency distribution. The opposite, a U value of 1, indicates a flat frequency distribution, where all the available units are emitted with the same frequency.

Barba (2012a) highlighted that the property that defines the descriptive class (lag, frequency, and threshold contingencies) is different from the property by which the functional class is measured (commonly U value). As a result, the differentiation process through which the functional class is shown to correspond more and more closely to the descriptive class is not demonstrated. We will add that, even if such a correspondence between the descriptive and the functional classes could be demonstrated, Catania’s third criterion above would not automatically be met. To illustrate the point, let us consider the case of “operant reinforcement of autonomic responses.” For example, reinforcers may be contingent on an increased heart rate (descriptive class) and, as a result, the heart rate may increase (functional class). Yet, the change in heart rate may depend on a change in other behavior, involving increased physical activity, which may be operant (e.g., Carroll & Rhys-Davies, 1979; Hatch & Gatchel, 1979). Thus, even the fact that a descriptive class is accompanied by a corresponding functional class is not sufficient as proof of an operant class or dimension. The assumed operant dimension may have come about as an indirect effect of experimenter-controlled contingencies.

How do we define a unit of behavior? After all, behavior is fluid and continuous, and not naturally divided into neat units (e.g., Epstein, 1991; Thompson & Lubinski, 1986). Skinner (1935) proposed to define units as classes by their function rather than by their appearance. Thompson and Lubinski (1986) stressed the importance of delineating units based on consistent, and not arbitrary, criteria. They asserted that the success of the scientific study of behavior relies on the way continuous behavior is divided into classes of replicable behavior units because, as Zeiler (1986) stated, using invalid units easily leads to confusion as to the significance and meaning of data. Even so, common units in most basic research experiments are selected by the experimenters based on topography. Although the previously mentioned light-matrix is no longer typically used in variability experiments, the most common descriptive unit is still sequences consisting of a fixed number of responses, usually on two operanda, L and R. If the sequence consists of four responses the number of possible different sequences is 16 (2^4^), and if the sequence consists of eight responses the number is 256 (2^8^). These sequences, when complying with the variability criterion, constitute the pool of responses that may be selected for a descriptive operant class, that is, the class of responses upon which reinforcement is contingent. Other important and common independent variables are (1) a forced IRT, (2) timeout, and (3) the probability of reinforcement. In spite of the complexity of these sequences as descriptive units, Cherot et al. (1996) stated simply that, “when reinforcement depends on a particular sequence of responses, the sequence comes to function as an operant unit” (p. 497). However, whether a contingency actually results in a corresponding functional class is not obvious. A reasonable alternative is that the resulting functional units in experiments relying on sequences as descriptive classes can be individual responses and/or combinations of different individual responses, not necessarily conforming strictly to the descriptive unit (e.g., Schneider & Morris, 1992).

Catania (1971) concluded that there is no reason to believe that a reinforcer only strengthens the last, contiguous response when several responses are followed by a reinforcer. However, as Zeiler (1977) argued, the mere occurrence of a predictable pattern is insufficient evidence for a unit. It is required that the unit itself is conditionable. Sequences can function as units, as several experiments have shown (Fetterman & Stubbs, 1982; Schneider & Davison, 2006; Schneider & Morris, 1992). These experiments, using both pigeons and rats, have concluded that sequences of two individual responses may form a functional unit, but for sequences containing more than two responses or two-response sequences with long intertrial intervals the results are either inconsistent or the functional unit has been found to be individual responses. Reid, Dixon, and Grey (2008) suggest that we differentiate between integrated behavioral units, also called chunks (Catania, 2013), and behavioral chains, because reinforcement is assumed to operate at different levels in integrated behavioral units and behavioral chains. In integrated behavioral units “reinforcement is presumed to operate at the level of the integrated unit, independent of its constituent responses” (Bachá-Méndez, Reid, & Mendoza-Soylovna, 2007, p. 7), whereas in behavioral chains it is presumed that each response is separately influenced by the reinforcer.

Catania et al. (2015) found that reinforcers affect earlier responses in addition to the last lever press or key peck, which is contiguous with the reinforcer. Their results made them conclude that time separating individual responses from the reinforcer was more important than intervening responses for weighting the contribution of each single response. A related question of importance when using sequences as units are whether these will change as a unit when the contingencies change or whether such a change will affect the different responses in the sequence differently. Catania (1971) concluded that “any account in terms of sequences of pecks as response-units must deal with the different effects of reinforcement on pecks at different positions in the sequence” (pp. 281–282). Reid (1994) addressed this issue with rats, using sequences consisting of three lever presses. When responding was stable he altered the criterion sequence, either changing the first lever-press (e.g., from LLL to RLL) or the last lever-press (e.g., from RLL to RLR) of the required sequence. A change in the last response led to faster acquisition of the new sequence than when the change was in the first response in the sequence. This result supports the hypothesis that differential reinforcement affects the responses closest to the reinforcer to a greater extent than responses more temporally distant from the reinforcer, even if steady-state behavior was obtained before changes were initiated. Reid’s result, in an experiment using fixed sequences, was replicated in a variability experiment by Peleg, Martin, and Holth (2017) using a Lag 5 contingency, with three-response sequences as units. In addition, Reid et al. (2008) interpreted data from Neuringer, Kornell, and Olufs’s (2001) experiments on variability and extinction, and their analysis suggested that the conclusion made by Neuringer et al. (2001) could adequately be explained by response-level processes instead of sequence-level processes. These results suggest that several variables may influence how reinforcement/nonreinforcement affects different positions of the responses in the sequences. Hence, variability within sequences may be induced by the differential temporary distance of the individual responses to the reinforcer. The result challenges the interpretation that a “no-memory,” endogenous stochastic process (Jensen et al., 2006; Neuringer, 1986, 2002; Page & Neuringer, 1985) is necessarily the major source of variable responding in a variability contingency.

In variability experiments it is not standard procedure to include pretraining on differentiation of sequences as units (see, e.g., Neuringer, 1991; Page & Neuringer, 1985). Some have included a few sessions on an FR 4 schedule (e.g., Doughty & Galizio, 2015), and some experiments have used multiple schedules with a vary and a repeat condition, where one sequence of four responses has been reinforced in the repeat condition (e.g., Cohen et al., 1990; Doughty & Lattal, 2001; Odum et al., 2006). Yet, there is little or no evidence to suggest that the commonly used descriptive units, sequences of four responses or longer, typically turn into functional units, and again, invalid units may lead to confusion when interpreting the data (Zeiler, 1986). Still, the common descriptive units in these experiments are sequences of four to eight lever presses or key pecks, always separated by a forced IRT, and the data analysis uses the relative frequency of each sequence, whereas data analysis on individual responses are usually not applied. This seems to constitute a major problem, as the question remains whether other types of data analysis, performed on individual responses, or combinations of responses, will lead to the same conclusions. As Shimp (2014) has written, “If aggregated responses were otherwise different, the aggregate would consist of apples and oranges, as it were, and their average would fail to reflect meaningful relative frequencies of either apples or oranges” (p. 230). Using sequences of responses on two identical operanda is, on the other hand, easy to program and execute. According to Neuringer (2012), the Lag *n* schedules are easy to program, and the use of them is in accord with Skinner’s (1956) informal principle that “some ways of doing research are easier than others” (p. 224). In applied research the descriptive unit is commonly not sequences but rather socially relevant units of responses, as in vocal responding (e.g., Koehler-Platten, Grow, Schulze, & Bertone, 2013; Lee, McComas, & Jawor, 2002; Susa & Schlinger, 2012), pointing at different drawn images (Heldt & Schlinger, 2012) or activity selection (Cammilleri & Hanley, 2005). An exception to this is Dracobly et al. (2017), who used sequences consisting of four different colored blocks as a unit. A Lag 4 schedule produced varied responding in most participants, but some maintained a higher-order stereotyped behavior. One possible explanation for this, suggested by the researchers, was that the children might have discriminated that the response consisted of four trials of sequences of four blocks. This exemplifies the problem of using complex descriptive units, even with humans. Could it be that the commonly used complex descriptive units, in the form of sequences, obscure response-level processes, because we do not know what the functional unit is, and therefore make moment-to-moment interpretation of the data more challenging?

In sum, it is still not clear what constitute functional units in these experiments. Whether calculated from individual responses or sequences consisting of several responses, the results for experiments using a variability contingency, do show variable responding. However, as the common analyses are conducted without consideration of potential functional units, reinforcement, and timeout, we question whether the statistical measures commonly used necessarily give a valid description.

## Molar and Molecular Interpretations of Variability

Shimp (2014) argued that behavioral variability has different meanings in molar and molecular analyses. Although the molar/molecular distinction may have different interpretations, a molar analysis is understood here as a statistical analysis of aggregated behavior, either between subjects or within subject over time, focusing on the strengthening effect of reinforcement. In contrast, a molecular analysis is considered to be a moment-to-moment analysis of ongoing behavior within a single subject, focusing on the shaping effect of reinforcement (Shimp, 2014). According to Shimp, the molar claim that aggregated free-operant behavior is under the control of its consequences, implies that there is no moment-to-moment shaping effect of individual patterns. In a molar perspective, therefore, moment-to-moment behavior “has to be emitted independently of momentary stimuli, independently of time since previous behaviors and events, and has to have sequential properties that can [be] described only in terms of random emission” (Shimp, 2014, p. 234). Shimp’s explanation of the molar interpretation of behavior exemplifies a standard way of interpreting variability data. Possible moment-to-moment shaping effects are difficult to search for, due to the large and complex units and the extended use if aggregated statistics, in addition, identifying the existence of these potential processes is commonly not a priority. In the few cases where shaping effects have been identified, researchers have tentatively suggested that the behavior may be a joint product of the molar variability-contingent reinforcement, and immediate consequences (Doughty & Galizio, 2015; Doughty & Lattal, 2001; Neuringer, 1991, 1992).

Both Sidman (1960) and Skinner (1976) argued that statistical analysis of aggregated data, even within an individual, can lead to loss of important information about the moment-to-moment behavior under study. Epstein (2014) suggested that there might be an orderliness in variable behavior, which sometimes seems random and unpredictable. He argued that the observed variability might be due to the methodological and statistical procedures used in these experiments, and that these statistical approaches obstruct a proper understanding of the behavior, because it is happening moment-to-moment. Epstein’s argument is perfectly aligned with Shimp’s (2014), who argued that the molar interpretation dismisses any shaping effect on the temporal organization of variable behavior and suggests that molecular behavior varies randomly with a constant probability over time. He concluded that there is little, if any, literature that supports this molar interpretation of molecular behavior and “a considerable literature that suggests it is incorrect” (Shimp, 2014, p. 224). Both Epstein (2014) and Shimp (2014) have argued that disregarding possible shaping and other moment-to-moment effects does not necessarily mean that there are no such effects. Doughty and Lattal (2001) also suggested that a molecular analysis of the serial order in variability experiments may show orderliness where molar statistical analyses, like the commonly used U value calculated over entire sessions, do not.

The few somewhat more molecular analyses of sequences performed in variability experiments, show that different variables at least sometimes have local effects. Neuringer (1991) analyzed the local effect that reinforcement and timeout had on sequences consisting of four responses. He found that if a sequence ended in reinforcement the operandum of the last individual response in the first sequence was likely to be repeated as the first response in the next sequence. On the other hand, if the previous sequence ended in a timeout, a switch to the other operandum was more likely. Neuringer’s (1992) results extended this finding to entire sequences, in an experiment where repetitive and varied sequences were reinforced according to different probabilities. Hence, if a sequence ended in reinforcement the entire four-response sequence was more likely to be immediately repeated, whereas a sequence ending with a timeout was less likely to be immediately repeated. Doughty and Lattal (2001) found response stereotypies for some of the birds in their variability experiment. One bird tended to peck the same key as the third response in the sequence, another bird would commonly start a sequence with a left key peck, and yet another subject often ended the sequences with a repetition.

Thus, analysis of moment-to-moment responses shows that even when a variability contingency is in effect, reinforcement increases the probability of a repetition, whereas timeout and delay of reinforcement decreases it. So, even if statistical analyses computed over entire sessions indicate that the data show variable behavior, more molecular analyses have found at least some order and predictability. It is important to note that even if “molecular” shaping effects within sequences show orderliness, or at least some orderliness, it does not necessarily entail that the responding is not variable. What it does entail, however, is that the responding is not necessarily unpredictable. Variability, uncertainty, and unpredictability sometimes seem to be used interchangeably (Jensen et al., 2006; Neuringer & Jensen, 2013). It is possible that the variable responding in variability contingencies is more predictable than standard interpretations of experimental results so far have suggested.

## Variability as a Derivative Process

In a variability contingency, such as a Lag 1 schedule, once a particular sequence has led to the reinforcer, that sequence is temporarily excluded from the descriptive operant and thus exposed to extinction until after another sequence has occurred. This is an example of a recency schedule, but frequency and threshold schedules function in a similar fashion, except that in these schedules the responses temporarily under extinction (or having a low probability of reinforcement), are not necessarily the last responses. Rather, it would be the most frequently emitted responses over some predetermined period. In a least-frequent schedule it would be all the other defined responses than the least frequently emitted response. Temporary exclusion of several sequences from the descriptive unit is common in these procedures. The critical point here is that the responses most recently emitted or most frequently emitted, are the responses under temporary extinction, and these most recently or most frequently emitted responses change continuously with responding (see also, Barba, 2012a; Machado, 1993). Thus, reinforcement selects responses or patterns of responses and maintains them, whereas local extinction induces variability. In a variability contingency, extinction is contingent on non-variability (i.e., repetition), so the more repetition of patterns the more extinction, which again produce more variability, which leads to reinforcement, leading again to more repetition, and so on.

We suggest that responses are repeated in a variability contingency, only to be extinguished when reinforcement is not forthcoming. The pattern of repeated responses would probably have been relatively easy to observe, at least in the first sessions of the experiments, had it not been for the elusive units in these experiments. The principle of reinforcement requires that the probability of a reinforced response will increase (e.g., Ferster & Skinner, 1957; Tatham, Wanchisen, & Hineline, 1993). In the first sessions, because the organism has no previous training with sequences as units, we can be confident that the sequence of four responses is not a functional unit, and repetition of the sequence would therefore not be expected. Thus, because reinforcement and extinction do not operate at the level of the observed descriptive unit, the use of these units makes the extinction of repeated patterns difficult to observe. This process will become even more difficult to observe when steady state is reached after several sessions, because the extinction process is known to speed up with repeated exposure (e.g., Clark & Taylor, 1960; Perkins & Cacioppo, 1950). The main difficulty with repetitive-response sequences as descriptive units is that they have no well-defined end—other than that forced by either reinforcement or time-out. Thus, exactly what is reinforced when a sequence produces a reinforcer is extremely hard to observe. A pattern of repetition of just-reinforced responses and a clear decrease in its frequency when not reinforced would be difficult to identify. However, when descriptive units consist of topographically distinct responses, there is evidence to suggest that such a pattern of repetition of the just-reinforced response and a decrease in this response in the absence of reinforcement is exactly what emerges (Holth, 2012).

Because they did not manage to teach the pigeons to repeat an eight-response sequence in a repetition component, Page and Neuringer (1985) excluded the possibility that extinction could be the major contributor to the variable behavior shown in their experiments. They concluded that it was unlikely that the pigeons could execute a specific eight-peck sequence and repeat it so that this sequence could then be exposed to extinction and a new sequence of eight pecks induced. We argue that this conclusion is valid only with respect to the entire response sequence as a unit. To the extent that only parts of the sequence, single responses, or possibly doublets are the actual functional unit, the intermittent extinction theory is not easily dismissed based on the findings mentioned above.

Neuringer and Jensen (2012) pointed out that neither variability nor changing levels of variability by themselves are sufficient to conclude that variability is an operant. An additional requirement is an “*independent* contingency-of-reinforcement control, i.e., independent of elicited or induced effects and independent of average values” (Neuringer & Jensen, 2012, p. 74). We have suggested that the yoke condition often claimed to show the direct function of a variability contingency on establishing variability as an operant dimension is not sufficient. An important consideration with the yoke condition is the removal of differential reinforcement specifically targeting unlikely responses found to increase during extinction (Neuringer et al., 2001). As explained by Machado and Tonneau (2012), when reinforcement is contingent on nonfrequent responses, a strong response (a response often or recently reinforced) will not be reinforced and will weaken. On the other hand, a response not often emitted or emitted a long time ago, and therefore not reinforced recently, is more likely to be emitted and reinforced, whereas more recently reinforced responses are under extinction. The differential extinction present in variability contingencies continuously weakens strong responses and differentially reinforces unlikely, rare responses evoked by the intermittent extinction. No such differential reinforcement of rare or unlikely responses is involved in the yoke procedure. Thus, the well-documented greater response variability during variability contingencies than during the yoke procedure seems fully explained by the fact that differential reinforcement of infrequent responses, hence differential extinction of frequent responses, is implicit in the variability contingency but not in the yoke procedure. When the variability contingency is lenient and the responses that satisfy the contingency are relatively simple, the functional operant class will correspond closely to the descriptive class. This correspondence will imply a higher-order stereotypy—or consistency—with repeated cycling through some of the different exemplars of the descriptive class (Abreu-Rodrigues et al., 2005). However, when the variability contingency is more stringent and/or the responses exposed to the contingency are more complex, stimulus control by previous responses is muddled, and variability results.

There is little doubt that induced variability is part of the variability observed in operant-variability studies (Morgan & Neuringer, 1990; Neuringer, 2012). In fact, no variability experiment to date has excluded the possibility of an induced variability effect inherent in extinction. Yet, as pointed out by Neuringer and Jensen (2012), variability as an operant dimension is what remains when such induced variability is subtracted. Interpreting the evidence to date, we suggest that a more productive and useful way to explain how a variability contingency engenders and sustains variable, random-like responding, is by a *dynamic process of intermittent conditioning and extinction of responses or response patterns*. When the descriptive operant consists of sequences, these patterns are not necessarily made up by the whole descriptive sequence, but likely by individual responses, doublets (e.g., a changeover from one operandum to another, or a stay at one operandum), triplets, and so on. Reinforcement continuously selects new patterns, induced by the differential extinction, and it also maintains responding. So, although reinforcement is necessary to maintain operant responding whether variable or not, it does not automatically follow that variability is directly reinforced. In short, it seems most satisfactory to consider the production of behavioral variability as a derivative, dynamic process, in accordance with Machado’s (1993) Frequency Dependent Selection and Barba’s (2015) Balance Hypothesis. Variability contingencies boil down to local periods of extinction, locally inducing variable behavior, and then to reinforcement of (a few or many) different responses.

## Challenges to the View of Variability as a Derivative Process

In the previously mentioned experiments by Neuringer (1986) human participants distributed two individual responses randomly according several statistical criteria. Thus, the results of that study may seem to strengthen the view of variability as a directly reinforced dimension. However, the mechanisms through which the results came about are difficult to analyze. Although the feedback in the form of performance graphs following each 100-response trial was called reinforcement (see also Neuringer and Jensen, 2013), that feedback likely had different functions. To some extent, the feedback may have functioned as negative reinforcement, resulting in participants avoiding certain types of feedback patterns. The maintenance of such behavior across a later period with no programmed feedback is hardly surprising (cf. Sidman, 1955). Perhaps more important was the potential role of the feedback as instruction governing behavior on succeeding trials. In Experiment 1, the feedback graph was accompanied by continuous suggestions regarding how behavior could be adjusted to satisfy the current statistical tests. Additional instructions accompanied successively added tests. Although instruction was reduced in Experiment 2, the experimenter still described the statistical randomness criteria as they were successively added. In addition, the experimenter described to each participant how different performance patterns might affect statistical evaluations at each step. Thus, the functions of different types of feedback as independent variables in Neuringer’s (1986) study are not clear (see also, Barba, 2012b).

High rates of reinforcement are sometimes seen in variability experiments, in particular where lenient or moderately lenient variability contingencies are used (e.g., Baumann, Abreu-Rodrigues, & da Souza, 2009; Doughty & Galizio, 2015, experiment 2). In some sessions rate of reinforcement has even reached a 100%, as in Page and Neuringer’s (1985) Experiment 6, a Lag 5 session where sequences of eight responses were the descriptive unit. This seemingly refutes the possibility that differential extinction may be the most important variable in these contingencies. Experimental results that may explain these high rates of reinforcement include Abreu-Rodrigues et al. (2005), who found that after extended exposure to a lenient and moderately lenient variability contingency pigeons showed efficient strategies of higher-order stereotypies in sequences of four key-pecks in Lag 1 and Lag 5 conditions. In the more stringent Lag 10 condition this stereotypy was not observed, and the rate of reinforcement was drastically lower. Another possible explanation was given by Machado (1997) who suggested that the variability of response sequences in variability contingencies may be an indirect effect of adjusting frequency of changeovers.

High rates of reinforcement and sessions without exposure to extinction have also been found in applied research (Lee et al., 2002; Lee & Sturmey, 2006). It is important to remember that most interventions in applied research commonly uses low lag schedules, and the resulting behavior is most often of the type that we previously called “variability where antecedents maintain discriminative control.” In these procedures, as previously mentioned, one type of response becomes an S^Δ^ for responding to the same type of response again, and an S^D^ for switching to another response. That some individuals, having been exposed to this contingency for some time, manage to respond in accordance with the contingency a 100% of the time is not surprising, and it does not refute the interpretation that extinction could have been the most important independent variable in the beginning of the intervention, when this behavior was acquired. Although appreciating that research on direct reinforcement of operant variability has been successfully applied in basic research, we would still argue that using the term direct reinforcement of variability (e.g., Dracobly et al., 2017; Lee et al., 2007; Miller & Neuringer, 2000) for these procedures may be misleading. In a lag schedule repetition is differentially extinguished whereas the variants that appear during extinction are differentially reinforced, as in any other procedure based on extinction and differential reinforcement of another behavior. For example, Betz, Higbee, Kelley, Sellers, and Pollard (2011) used extinction and a DRA procedure and wrote: “To promote extinction-induced variability, reinforcement is typically provided the first time a particular response topography is emitted, after which that response form is no longer reinforced” (p. 357). The only difference between these two procedures is that in an extinction and DRA procedure the behavior under extinction is consistent throughout the intervention, whereas in a lag schedule, the behavior under extinction continuously changes with responding. Because lag schedules supposedly directly reinforce variability, they have been presented as a superior alternative, and it has been suggested that they will not result in problematic side effects, such as extinction-induced aggressive outbursts (Miller & Neuringer, 2000). This seems misleading and we would argue that extinction of repetition, and intermittent reinforcement of different responses would be a more appropriate characterization.

## Conclusion

Variability is very important in behavior analysis and yet disproportionately sparsely researched. Research by Neuringer and colleagues has contributed tremendously to the field, showing that certain contingencies reliably produce behavioral variability. Even so, the prevailing view of behavioral variability as directly reinforced and as an operant dimension seems incongruous with several empirical findings. In order to determine any direct contribution to variability by reinforcement, experiments in this field need to be conducted without the known additional variability-inducing independent variables typically present (e.g., the forced IRT) and by establishing reliable behavioral units. In addition, it is important to realize that the extinction component cannot be removed from any differential reinforcement contingency and to acknowledge the variability-inducing effect of this component—which leads to different responses being exposed to immediate reinforcement. There is no doubt that variability can be increased with a variability contingency, and as such the contingency may be defined as a reinforcement procedure (Machado, 1994). What we question is whether experiments so far have shown that this is best considered as direct reinforcement of variability. Neuringer (1992) made the point that behavior may be jointly controlled by both molar and immediate consequences. The question raised here is whether even the behavioral variability attributed to molar contingencies can be attributed to more local contingencies.

Interpretations of steady-state responding in variability experiments have not typically involved a discussion of the importance of differential extinction as necessarily embedded in variability contingencies. Yet it seems unthinkable that such extinction plays no significant part in inducing variability. Most other operant dimensions do not have this problem to the same extent, at least not in steady state. Rather than direct reinforcement of variability, “direct extinction of repetition” seems central to the emerging variability. This interpretation of data from variability experiments should satisfy Occam’s razor better than the notion of operant variability because it explains the variable responding as a derivative process, it does not lead to conclusions that oppose well-known behavior-analytic principles, and neither does it presume the existence of an internal stochastic-generator. If extinction of repetition, or in particular of repeating patterns, is central in producing variable behavior, the fact that longer sequences conform to the contingency faster and more closely makes perfect sense. The harder a pattern would be to repeat, the faster it would disappear. Also, the forced IRTs probably make it less likely that the sequences used as descriptive units form functional units. This will also result in the pattern being less likely to be repeated.

An important additional concern is that the descriptive units in variability studies (i.e., sequences) have been unjustifiably complex. Also, the functional unit(s) produced in most of the experiments conducted to date on behavioral variability have been fuzzy at best, and it is essential to be able to isolate and calculate data from these functional units. Our last concern is the use of the yoke procedure as a control condition for differential extinction. A continued neglect of these important procedural issues will continue to cause problems in the interpretation of variability data. If the issue of whether behavioral variability should be considered as a directly reinforced operant dimension of behavior can be settled at all, perhaps of crucial importance is the development of different experimental procedures. We believe that persistent use of sequences as descriptive units will continue to impede further understanding. Studying distinct topographies, rather than sequences consisting of several responses to a limited number of operanda, will remove some of the variability-inducing variables. It should also make the descriptive unit more distinct, and functional units easier to delineate, thereby making it easier to identify possible local shaping effects in future variability experiments. This may lead to results revealing that the resulting variable responding may not be as unpredictable as previously suggested.

## Appendix

The common formula for calculating the U value:

$$ U=-{\sum}_{i=1}^N\frac{Rf_i\times {\mathit{\log}}_2\left({Rf}_i\right)}{{\mathit{\log}}_2(N)} $$

Where *N* equals the number of available categories, and Rf the relative frequency of each category.
